# Identifying Multi-Omics Causers and Causal Pathways for Complex Traits

**DOI:** 10.3389/fgene.2019.00110

**Published:** 2019-02-21

**Authors:** Huaizhen Qin, Tianhua Niu, Jinying Zhao

**Affiliations:** ^1^Department of Epidemiology, College of Public Health and Health Professions and College of Medicine, University of Florida, Gainesville, FL, United States; ^2^Department of Global Biostatistics and Data Science, Tulane University, New Orleans, LA, United States; ^3^Department of Biochemistry and Molecular Biology, Tulane University School Medicine, New Orleans, LA, United States

**Keywords:** associations, causations, transcriptomics, proteomics, data integration, systems biology

## Abstract

The central dogma of molecular biology delineates a unidirectional causal flow, i.e., DNA → RNA → protein → trait. Genome-wide association studies, next-generation sequencing association studies, and their meta-analyses have successfully identified ~12,000 susceptibility genetic variants that are associated with a broad array of human physiological traits. However, such conventional association studies ignore the mediate causers (i.e., RNA, protein) and the unidirectional causal pathway. Such studies may not be ideally powerful; and the genetic variants identified may not necessarily be genuine causal variants. In this article, we model the central dogma by a mediate causal model and analytically prove that the more remote an omics level is from a physiological trait, the smaller the magnitude of their correlation is. Under both random and extreme sampling schemes, we numerically demonstrate that the proteome-trait correlation test is more powerful than the transcriptome-trait correlation test, which in turn is more powerful than the genotype-trait association test. In conclusion, integrating RNA and protein expressions with DNA data and causal inference are necessary to gain a full understanding of how genetic causal variants contribute to phenotype variations.

## Introduction

**Figure 1:**
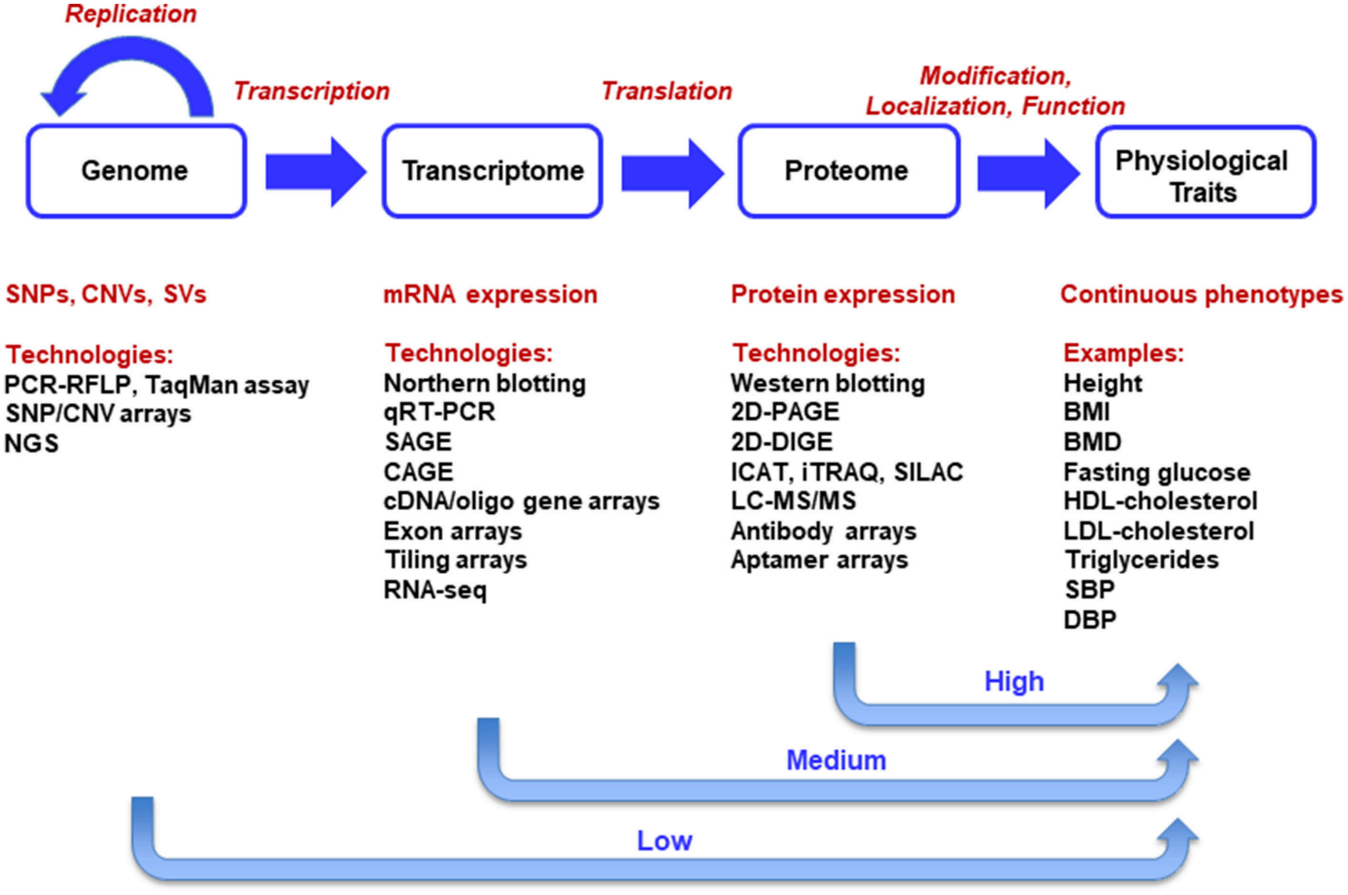
Integration of Multi-Omics Data Sets in the Context of Central Dogma. The central dogma of molecular biology delineates a unidirectional causal flow from genome to transcriptome, proteome, and phenome. Systems biology, the integration of multi-omics technologies, aims primarily at the universal detection of causal genes, mRNAs, proteins, and causal pathways for phenotypes in a holistic manner.

The central dogma of molecular biology, as first proposed by Francis Crick (Crick, 1958, 1970), describes the transfer of sequence information during DNA replication, transcription into RNA and translation into amino-acid chains forming proteins. There are only ~23,500 predicted protein-coding genes in humans. Such genes constitute only ~2% of human DNA sequence. Genetic studies of thousands of single-gene disorders have revealed a large set of mutations in protein-coding regions, which appears to support the central dogma that the major output of the genome is protein (Plomin and Davis, 2009). Advanced multi-omics technologies have led to generation of genome-scale data sets at DNA, RNA, and protein levels. The multi-level data sources are illustrated in the context of the central dogma in Figure 1. At DNA level, genomic data uncover the information stored in the genomes of organisms. Variations at DNA level in populations include single nucleotide polymorphisms (SNPs), copy number variations (CNVs), and structural variations (SVs) (Koyutürk, 2010).

Identification of genetic causal variants for complex traits poses dramatically greater challenges than that for Mendelian traits, due to low penetrance, variable expressivity and pleiotropy, epistasis, and locus heterogeneity (Nadeau, 2001; Glazier et al., 2002). Klein et al. (2005) pioneered a genome-wide association (GWA) study and identified a SNP in *CFH* gene on age-related macular degeneration. GWA studies have proven successful to identify susceptibility genetic variants for a range of complex traits (Christensen and Murray, 2007; Stranger et al., 2011; Evangelou and Ioannidis, 2013; Manolio, 2013). To date, at least 11,912 trait-associated SNPs from 1,751 curated publications have been reported at NHGRI Catalog of Published GWA studies (http://www.genome.gov/gwastudies/) (Welter et al., 2014). DNA variants for a plethora of physiological traits have been revealed by GWA studies, e.g., height (Weedon et al., 2008), body mass index (Monda et al., 2013), bone mineral density (Estrada et al., 2012), lipid levels (Willer et al., 2008), and hypertension (Kato et al., 2011). In past decade, researchers have also extended genetic association studies to rare genetic variants for a range of complex diseases. Typical examples include extreme lipoprotein cholesterol levels (Cohen et al., 2004; Haase et al., 2012), obesity (Ahituv et al., 2007; Meyre et al., 2009; Coassin et al., 2010), type 1 diabetes (Nejentsev et al., 2009), bone mineral density (Kung et al., 2010; Duncan et al., 2011). However, for a complex trait, the identified genetic variants only account for a small portion of phenotypic variation (Ruiz-Narváez, 2011).

A majority (88%) of trait-associated SNPs were found to reside in intergenic or intronic regions (Hindorff et al., 2009), suggesting that non-coding regions of the genome are responsible for most of the disease risk (Kung et al., 2010; Duncan et al., 2011) and some of this risk is likely to act through gene regulation (Bossé, 2013). Based on the central dogma of molecular biology, it is mechanistically very well understood how a gene get transcribed, how an mRNA get processed and sequentially translated into amino acid chains at the ribosome and subsequently fold into functional proteins. Recently, remarkable advances of RNA-seq and mass spectrometry technologies have rapidly improved global identification, quantification, and analysis of transcriptome and proteome in same biological samples. Correlations between mRNA and protein abundances turn to be much stronger than that between genotype and trait. In bacteria and eukaryotes, they often show a squared Pearson correlation coefficient of ~0.40, which implies that ~40% of the variation in protein abundance can be explained by knowing mRNA abundances. Emerging evidence shows that many regulatory mechanisms occur after mRNAs are made, and proteins exhibit a larger dynamic range of concentrations than do transcripts (Jacobs et al., 2005; Vogel et al., 2010; Gonzàlez-Porta et al., 2013; Schwanhäusser et al., 2013). A combination of post-transcriptional, translational and degradative regulation, acting through miRNAs (Mukherji et al., 2011) or other mechanisms to fine-tune protein abundances to their preferred levels. For example, miRNAs have been found to fine-regulate protein expression levels, rather than to cause large expression changes (Baek et al., 2008; Selbach et al., 2008). The combination of association studies on RNA and protein expressions allows systematic identifications of expression quantitative trait loci (eQTLs) and protein QTLs (pQTLs).

Single-platform studies, although popular, often neglect significant amount of genomic information. Under the central dogma, the genetic variant is most remote from the physiological trait; and the transcriptional and translational processes can dramatically attenuate the genetic effect of the genetic variant. Even if the sample size reaches several tens of thousands, the power is very limited to detect common genetic variants of modest effect sizes (Galvan et al., 2010). For example, Panagiotou et al. (Panagiotou et al., 2013) depicted the relationship between the sample size and the number of trait-associated loci of genome-wide significance (*P* < 5 × 10^−8^) in GWA studies of height, lipid levels, and blood pressure. Conventional genetic association studies ignore the mediate causers and the unidirectional causal pathways; and the genetic variants identified are not necessarily genuine causal variants, but are only in close physical proximities to them (Kingsley, 2011). Therefore, it is necessary to integrate multi-omics data and model a unidirectional causal graph. In this article, through extensive analytical explorations under the central dogma, we demonstrate that proteome data have the greatest potential to enhance our understanding of physiological traits, because protein is a direct causer for physiological trait and has a stronger correlation with trait than either genotype or transcriptome. We provide power and sample size analyses under extreme phenotype sampling (EPS) and random sampling schemes. EPS has been widely employed to detect genetic causal variants for complex diseases (Lander and Botstein, 1989; Abecasis et al., 2001; Xiong et al., 2002). Herein, we extend this sampling strategy to a multi-omics setting. The results would be helpful to design cost-effective multi-omics studies as well as to develop novel multi-stage causal association inference methods.

## Methods

### Multi-Omics Causal Model (MCM)

**Figure 2:**
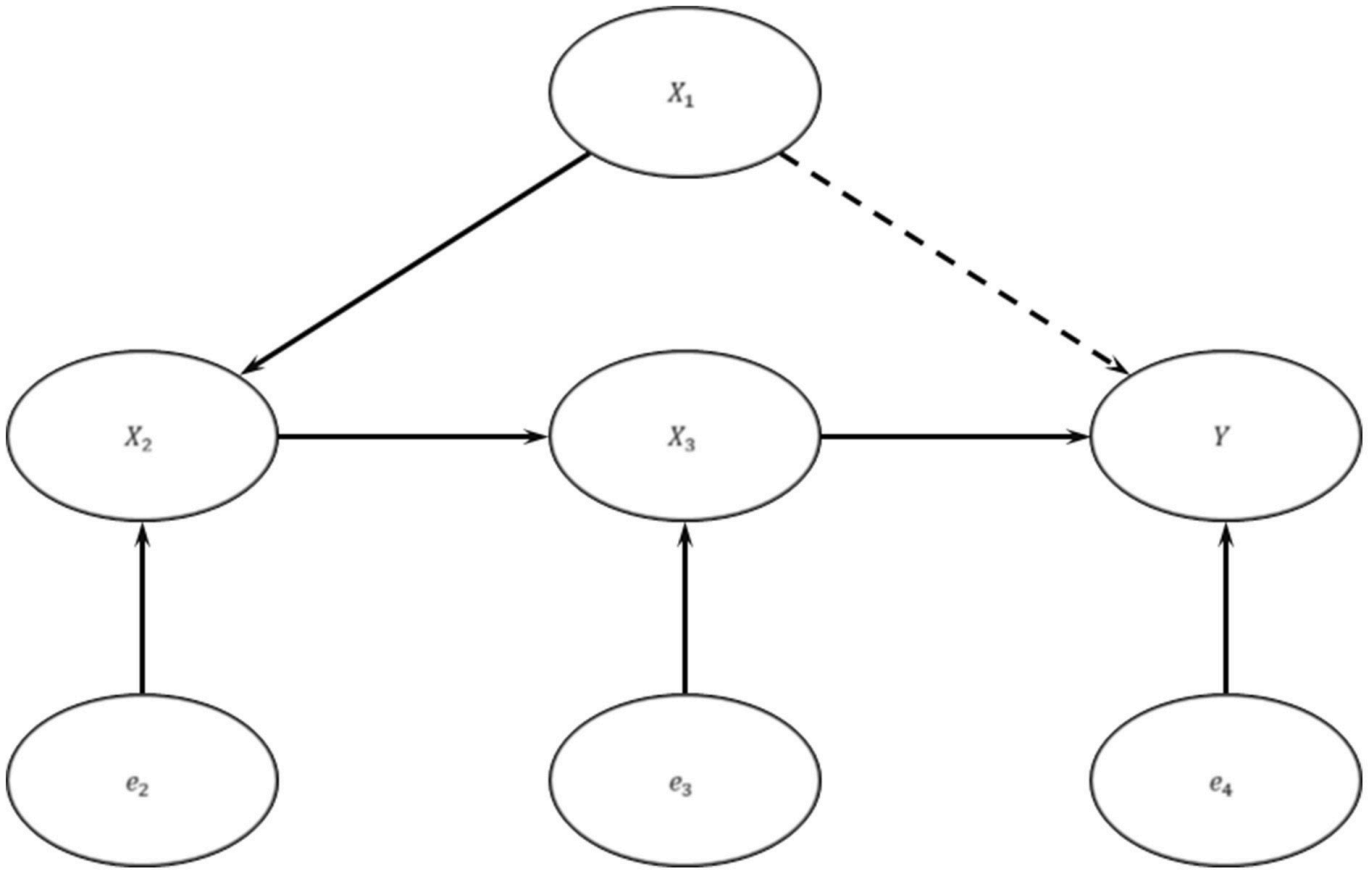
A Multi-omics Causal Model. In this system, *X*_1_ is the copy number of the minor allele at a causal SNP, *X*_2_ is RNA expression level, *X*_3_ is protein expression level, *Y* is trait value, and the *e*'s are exogenous errors. The exogenous errors are mutually independent with each other.

A mediate causal graph may be suitable to model the unidirectional causal flow delineated by the central dogma of molecular biology. Assume the genuine data generating model is as depicted in Figure 2: {*Y* = *X*_3_β_3_ + *e*_4_,*X*_3_ = *X*_2_β_2_ + *e*_3_*X*_2_ = *X*_1_β_1_ + *e*_2_}, where *X*_1_ is the genotypic score (copy number of the minor allele) at a causal SNP, *X*_2_ is RNA expression, *X*_3_ is protein (PRT) expression, and *Y* is trait value of interest. Let exogenous errors *e*_2_, *e*_3_, and *e*_4_ be independent.

Let $\sigma_{\cdot }^{2}$denote the variance of a variable. Let $r_{1}^{2}=\beta_{1}^{2}\sigma_{X_{1}}^{2}/\sigma_{X_{2}}^{2},\;r_{2}^{2}=\beta_{2}^{2}\sigma_{X_{2}}^{2}/\sigma_{X_{3}}^{2},$ and $r_{3}^{2}=\beta_{3}^{2}\sigma_{X_{3}}^{2}/\sigma_{Y}^{2}.$ Then the heritability of the causal SNP is $\;h^{2}=r_{1}^{2}r_{2}^{2}r_{3}^{2}$. In the numerical explorations, the minor allele frequency (MAF) of the causal SNP was fixed at *p* = 0.25, $r_{1}^{2}$ ranged from 0 to 15% (Schadt et al., 2003; Morley et al., 2004; Dimas et al., 2009; Webster et al., 2009; Bryois et al., 2014), $r_{2}^{2}$ ranged from 0 to 50% (Guo et al., 2008; de Sousa Abreu et al., 2009; Lundberg et al., 2010; Vogel et al., 2010). Protein studies are based on extreme phenotype sampling (EPS) with certain truncation levels α (e.g., α = 0.1~0.2). In other words, an identical number of samples are drawn from top and bottom 100α% tails of trait distribution, respectively. Reported fold changes between two tails of protein intensity ranged from 1.1 to 5 (Fields, 2001; Selbach et al., 2008; Cairns et al., 2009; Qiu et al., 2012). Under the MCM and EPS with a truncation level α = 0.2, a 5-fold change in protein intensity is equivalent to $r_{3}^{2}=34\%$ (Appendix A). Hence, we varied $r_{3}^{2}$ from 0 to 34%, and varied the SNP heritability level *h*^2^ from 0.0% to 2.5% for numerical explorations.

### Working Models and Null Hypotheses

Under the MCM, the genotype-phenotype association test can be investigated under a simple additive genetic model (AGM), see Appendix A. To be specific, we rewrite the MCM as

(1) $$\begin{array}{r} \text{SNP}:Y=X_{1}\beta_{1}\beta_{2}\beta_{3}+\left(\beta_{2}\beta_{3}e_{2}+\beta_{3}e_{3}+e_{4}\right). \end{array}$$

Under this AGM working model, we test for *H*_0,SNP_ : β_1_β_2_β_3_ = 0. Meanwhile, the tests for the associations between a mediate causal variable and the phenotype can be investigated under respective single mediate causal variable models (SMCVMs). To test for RNA-phenotype association, we rewrite the MCM as

(2) $$\text{RNA}:\;\left\{ \begin{array}{l} Y=X_{2}\beta_{2}\beta_{3}+(\beta_{3}e_{3}+e_{4}),\\ X_{2}=X_{1}\beta_{1}+e_{2}. \end{array}\right.$$

Under this SMCVM model, we test for *H*_0,RNA_ : β_2_β_3_ = 0. To test for protein-phenotype association, we rewrite the MCM as

(3) $$\text{PRT}:\;\left\{ \begin{array}{l} Y=X_{3}\beta_{3}+e_{3},\\ X_{3}=X_{1}\beta_{1}\beta_{2}+\left(e_{2}\beta_{2}+e_{3}\right). \end{array}\right.$$

Under this working model, we test for *H*_0,PRT_ : β_3_ = 0. The PRT and RNA models share one common indirect causal variable (SNP genotype *X*_1_) and have respective mediate causal variables. In the PRT model, protein expression *X*_3_ is taken as the mediate causal variable; and in the RNA model, RNA expression *X*_2_ is taken as the mediate causal variable.

### Sampling Designs and Association Tests

Under simple random sampling (SRS), the classical *t* test for slope is suitable for the association tests. Due to prohibitive experimental costs, however, extreme phenotype sampling (EPS) is often adopted for cost-effective multi-omics studies. In protein studies, for example, EPS is often utilized to reduce experimental costs and maintain statistical power of the two-sample *t*-tests. In EPS protein studies, fold changes in intensity of promising proteins are often reported. From major journals, however, we have not identified protein studies that reported correlation coefficient between promising proteins and phenotypes. In Appendix A, we establish theoretical foundation to numerically evaluate powers of the SRS and EPS based *t*-tests for identifying the three kind of associations. In addition, we establish a method that converts a given fold change to corresponding correlation coefficient under the MCM.

### Software Package

Under the MCM, a freely available R package is developed for implementing numerical computations and graphical illustrations (https://github.com/HuaizhenQin/MCM/). It consists of R functions for multi-level power analyses and sample size evaluations under SRS and EPS. The function MCVM.Power(.) compute powers whereas the function MCVM.n(.) determines sample sizes to reach certain powers of level-specific tests under each sampling design. Both of the functions call corresponding functions for computing the non-centrality parameters. All the functions adopt fast and stable numerical algorithms without using any resampling techniques. The package is particularly useful for designing multi-omics studies and analyzing large-scale multi-omics data from such studies.

## Results

In numerical power and sample size analyses, we use the nominal significance level of 2.5e-6 to depict relative power and sample size patterns of the widely used *t-*test under different sampling schemes. This nominal significance level is suggested in Goldstein et al. (2013) and often used for genome-wide gene-based association tests, because there are approximately 20,000 protein-coding genes in the human genome (Dunham, 2018).

### Numerical Power Comparisons

**Figure 3:**
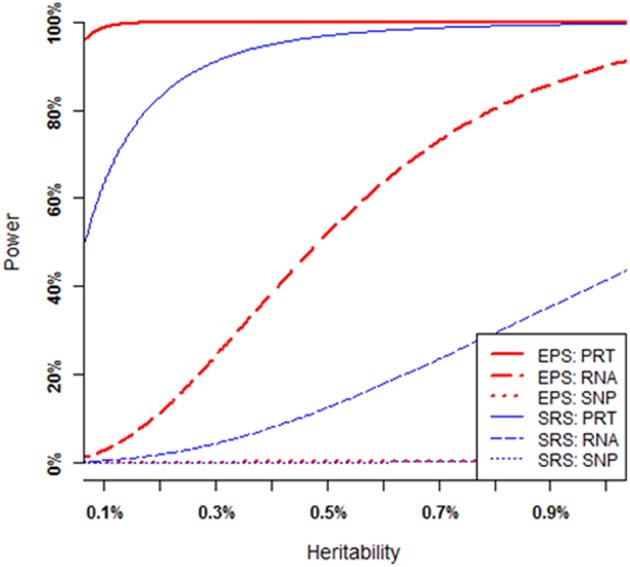
Power comparison at various heritability levels. Each overall heritability level of the causal SNP in the MCM is determined by a set of mediate effect sizes. All curves are numerically computed by setting minor allele frequency at *p* = 0.25, nominal significant level at 2.5 × 10^−6^, and total sample size at *n* = 200. The truncation level is set at α = 0.2 in the EPS.

As illustrated by Figure 3, the power of each strategy increases when the heritability of causal SNP increases (i.e., all the mediate correlations increase), provided that all other parameters (i.e., MAF of the causal SNP, nominal significance level, and sample size) are fixed (see Appendix B for a theoretical proof). Under SRS, the PRT test (blue solid curve) appears strikingly more powerful than the RNA test (blue dashed curve), and the RNA test appears strikingly more powerful than the SNP test (blue dotted line). The SRS-based single SNP test has little power to detect a genuine genotype-phenotype association over a large range of heritability levels. Under EPS, the PRT test (red solid curve) appears strikingly more powerful than the RNA test (red dashed curve), and the RNA test appears strikingly more powerful than the SNP test (red dotted curve). The EPS based single SNP test still has little power to detect the genuine genotype-phenotype association over the large range of heritability levels.

**Figure 4:**
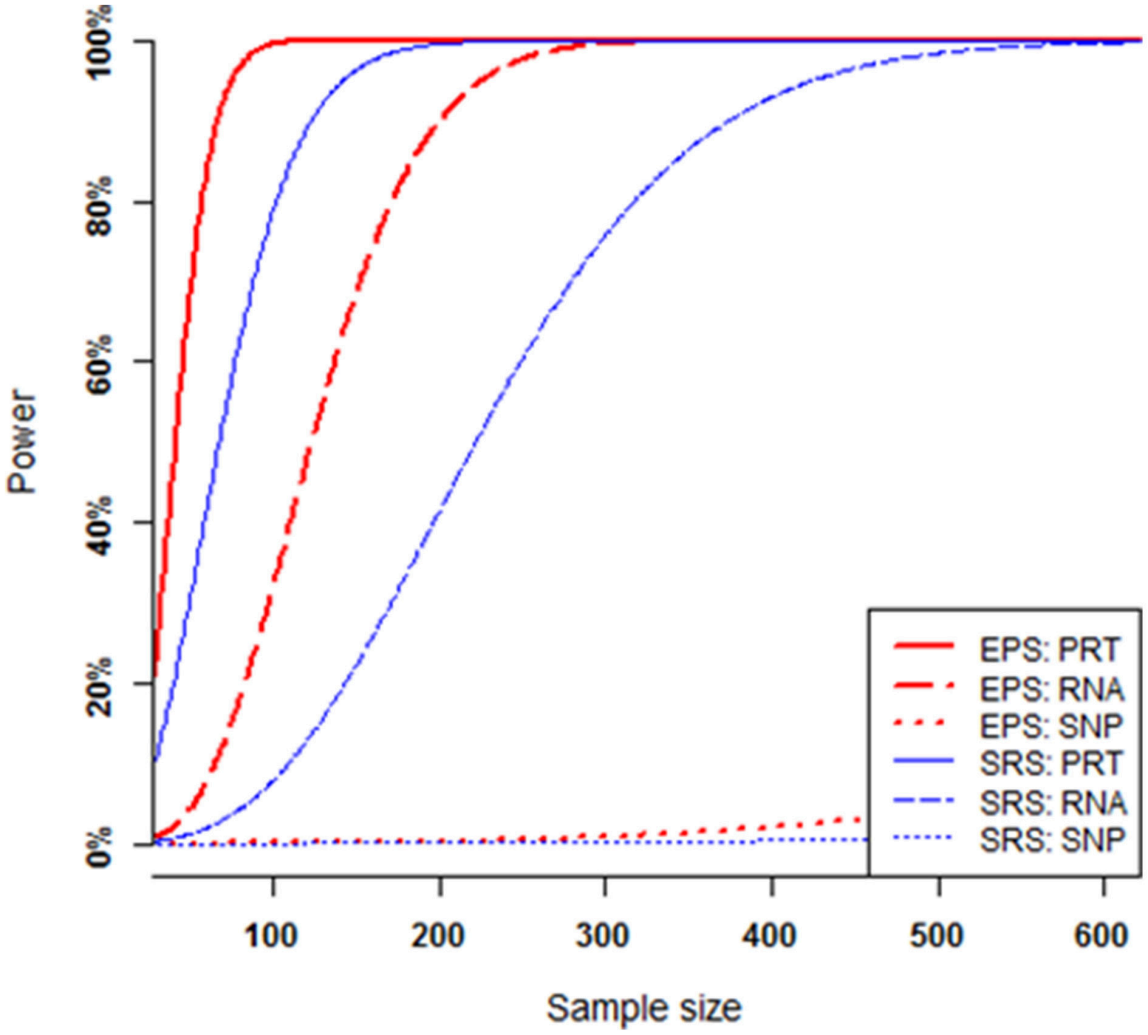
Power comparison at various sample sizes. All curves are numerically computed at nominal significant level 2.5 × 10^−6^ for a fixed SNP heritability level *h*^2^ = 1.0%, which is determined by a set of mediate effect sizes (β_1_ = 0.5744, β_2_ = 0.7183, β_3_ = 0.4564) and minor allele frequency *p* = 0.25. The truncation level is set at α = 0.2 in the EPS.

The power of each test increases when the sample size increases, provided that all other parameters (i.e., all the mediate correlations and hence the SNP's heritability, minor allele frequency at the causal SNP, and nominal significance level) are fixed (Figure 4). Under SRS, the PRT test appears strikingly more powerful than the RNA test, and RNA test appears strikingly more powerful than the SNP test. The SRS-based single SNP test has little power to detect the genuine genotype-phenotype association over the range of sample sizes. Under EPS, the PRT test appears more powerful than the RNA test, and RNA test appears more powerful than the SNP test.

For a given set of non-zero effect sizes in the MCM (Figures 3, 4), the EPS may provide much stronger evidence than does the SRS to reveal genuine associations between study phenotype and PRT expression (red vs. blue solid curves), RNA expression (red vs. blue dashed curves) and SNP genotype (red vs. blue dotted curves).

### Sample Sizes Required to Achieve a Certain Power

**Figure 5:**
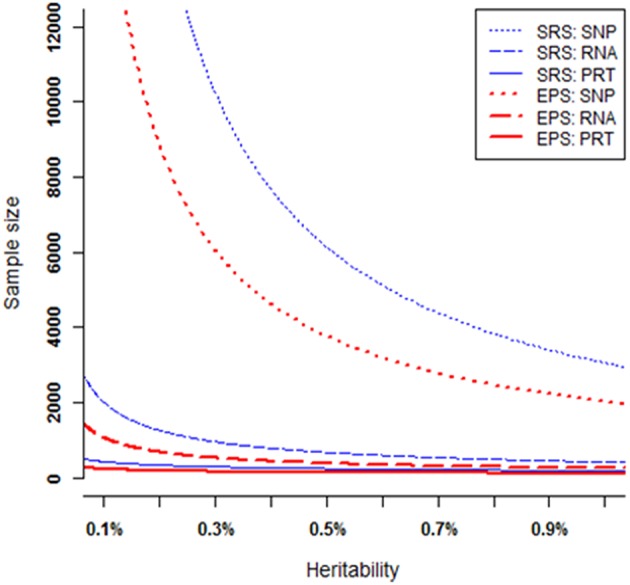
Sample sizes required to achieve 80% power. All curves are numerically computed by setting minor allele frequency at *p* = 0.25, nominal significant level at 2.5 × 10^−6^, and fixing power at 80%. The truncation level is set at α = 0.2 in the EPS.

At each heritability level *h*^2^ > 0, to achieve 80% power, these six strategies require very different samples sizes (Figure 5). Under both EPS and SRS, the SNP test requires the largest sample sizes, the RNA test requires much smaller sample sizes, followed by the PRT test. For a given heritability level, the SRS-based SNP test requires strikingly larger sample sizes than the other five strategies; and the EPS-based PRT test requires the smallest sample sizes among all the six strategies. The smaller the heritability level, the larger the difference between sample sizes required by the SRS-based and the EPS-based SNP tests. This statement also applies to the RNA and PRT tests. Table 1 lists the ranges in sample sizes of all these six strategies when SNP heritability varies from 0.1% to 1.0%.

**Table 1:** Ranges in sample size to achieve 80% power.

**Sampling**	**SRS**			**EPS**		
**Test**	**SNP**	**RNA**	**PRT**	**SNP**	**RNA**	**PRT**
*h*^2^ = 0.1%	30,409	1,540	245	16,129	795	127
*h*^2^ = 1.0%	3,055	317	102	1,744	172	57

## Discussion

“Omics” technologies have advanced rapidly in the past decade. Systems biology, the integration of multi-omics technologies, aims primarily at the universal detection of genes (genomics), mRNAs (transcriptomics), proteins (proteomics) and metabolites (metabolomics) in a holistic manner. Separate omics technologies and systems biology have generated and will continue to generate huge amounts of high dimensional multilevel data. The central dogma of molecular biology (Crick, 1958, 1970) delineates a unidirectional causal flow from DNA to mRNA, protein, and metabolite (physiological trait). Separate single-platform correlation tests are useful to understand causes of phenotype variation. We theoretically compared three single-platform tests under both random sampling and extreme phenotype sampling scenarios. The proteome-trait correlation test is more powerful than transcriptome-trait correlation test, which in turn is more powerful than genotype-trait association test. The sample size required to detect a causal gene is the smallest at proteomics level and the largest at the genomics level. A direct relationship between protein expression profiles and physiological traits implies that a smaller sample size can yield more meaningful insights than relating RNA expression profiles with traits.

RNA and protein expression levels can be mapped to chromosomal loci to identify functional DNA variants of a physiological trait. RNA and protein expression levels can be considered as intermediate phenotypes, as DNA variations contribute to the physiological trait by perturbing RNA and protein expressions (Civelek and Lusis, 2013). Gene expression levels are highly heritable (Morley et al., 2004; Lappalainen et al., 2013), and specific genetic variants that influence gene expressions are known as eQTL. Multiple studies have provided strong evidence that GWA signals are enriched with eQTLs in a tissue-specific manner (Dimas et al., 2009; Nicolae et al., 2010), highlighting their utility in understanding the mechanisms underlying GWA hits. Many resources, including online databases such as GeneVar (Yang et al., 2010), are now available for eQTL analyses. It is estimated that 50–90% of eQTLs are tissue-dependent (Dimas et al., 2009; Nica et al., 2011), and trait-associated variants tend to exert more tissue-specific effects (Fu et al., 2012; Brown et al., 2013). Most identified eQTLs are cis-acting, arbitrarily defined as regulation of genes within 1 Mb, given that their effect sizes are usually relatively large and can be detected with smaller sample sizes (Cheung and Spielman, 2009). However, genetic variants can also affect the expression of genes that reside further away or are on different chromosomes (trans-eQTL) (Westra et al., 2013), but the effect sizes of trans-eQTLs are generally small, and they require larger sample sizes to detect them (Cookson et al., 2009; Grundberg et al., 2012). As a result, the number of reported trans-eQTLs has remained small (Heinig et al., 2010; Consortium, 2011; Fehrmann et al., 2011; Innocenti et al., 2011; Fairfax et al., 2012). For example, the first GWA study of asthma was published in 2007 by the GABRIEL consortium (Moffatt et al., 2007), in which 317,000 SNPs in 994 patients with childhood-onset asthma and from 1243 nonasthmatics were genotyped using family and case–control panels. This association was then replicated in 2320 individuals from a cohort of German children and in 3301 individuals from the British 1958-birth cohort. The 17q21 region is the most consistent locus associated with asthma. Further, the most compelling eQTL associated with GSDMA expression in the lung tissues was found to reside in the same locus, suggesting that the risk allele at this locus mediate its effect by modulating GSDMA expression. The strongest eQTL SNP on 17q was rs3859192 located in intron 6 of GSDMA, which is associated with asthma (Moffatt et al., 2007, 2010). This example demonstrates the value of eQTLs (lung-specific) to refine (i.e., to fine-map) previous GWA hits for asthma. Shared eQTLs across multiple cell types and tissues also have larger effect sizes and tend to cluster around the transcriptional start site (TSS) (Dimas et al., 2009; Grundberg et al., 2012). In contrast, cell- and tissue-specific eQTLs have smaller effects and are more widely distributed around the TSS. The directions of the allelic effects for eQTLs shared in different cell types are usually consistent (Dimas et al., 2009), but cell type-dependent and tissue-dependent direction effects were also observed (Fairfax et al., 2012; Fu et al., 2012).

For protein-coding genes, functionally important changes in mRNA expression are expected to be reflected by corresponding protein changes. However, a weak correlation between transcript and protein levels in yeast shows that various other mechanisms of post-transcriptional regulation can lead to changes in protein abundance in the absence of a corresponding transcript effect (Gygi et al., 1999). Variation in protein expression levels have recently been shown to be heritable (Wu et al., 2013; Parts et al., 2014). In humans, pQTL mapping has lagged behind eQTL mapping. To date, only a few studies have explored association between SNPs and protein abundance levels (Lourdusamy et al., 2012; Hause et al., 2014). Those pQTLs that overlap with SNPs associated with physiological traits support previously identified mechanistic relationships and provide testable hypotheses of functional relationships. pQTL analyses may be helpful for gaining additional mechanistic insights into molecular underpinnings of physiological traits that is separate from eQTLs. For example, rs3865444 on chromosome 19q13.3 is strongly associated with Alzheimer's disease (AD) in a meta-analysis of several case–control studies (OR = 0.91, *P* < 1.9 × 10^−9^). The effect allele A of rs3865444 reduces the protein abundance of CD33 (beta = 20.45, FDR < 5.06 × 10^−9^) indicating that this pQTL might influence AD susceptibility through a mechanism of altered protein abundance. CD33 is a member of sialic acid-binding immunoglobulin-like lectin (Siglec) family, which regulates functions of cell in the innate and adaptive immune systems (García-Domingo et al., 1999). Thus, discoveries of eQTLs and pQTLs across multiple populations, cell types, and tissues will facilitate the identification of regulatory variation in complex traits and diseases.

Pairwise association studies often neglect a significant amount of causality information. Under the central dogma, the DNA variant is most remote from the trait; and as we illustrated, the transcriptional and translational processes can dramatically attenuate the association between the DNA variant and the trait. In practical scenarios, inconsistent and/or non-replicable findings among separate GWA studies are quite common, which posing the critical question as to how to properly interpret the incongruences of these results. Meta-analysis is a popular tool for combining multiple independent genetic association studies to identify associations with small genetic effect sizes. In the presence of genetic heterogeneity, interpreting the meta-analysis results is an important but often difficult task (Han and Eskin, 2012). Indeed, human disease is characterized by marked genetic heterogeneity, far greater than previously anticipated (McClellan and King, 2010). Such heterogeneities can greatly reduce the power of conventional association methods. Through extensive simulation studies, Pei et al. (2014) demonstrated that (i) in the presence of between-study heterogeneity, the true genetic effect might be diluted, and meta-analysis (even with the random-effects model) may particularly introduce elevated negative rates, and (ii) replicability between meta-analyses and independent individual studies is limited, and thus inconsistent findings are not unexpected. The presence of a substantial between-study heterogeneity could lead to a power loss in meta-analyses, implying that aggregating genetically heterogeneous samples into a meta-analysis may reduce power. Meta-analyses should not and cannot be used as a gold standard to evaluate the results of individual GWA studies (Liu et al., 2013). Conventional genetic association studies ignore the essential mediate causers (RNA, protein) and the unidirectional causal pathway. Thus, such studies are often underpowered, and may not necessarily discover genuine causal variants.

The central dogma of molecular biology indicates that the transcription of mRNA from DNA and subsequent translation of mRNA into protein transform genetic blueprints into cellular functions (Crick, 1958, 1970). However, epigenetic factors represent an additional layer of complexity to our understanding of gene regulation. Epigenetic changes, i.e., reversible, heritable changes in gene regulation that occur without a change in DNA sequence, include DNA methylation and histone modification (Baccarelli and Bollati, 2009), as well as microRNA and long non-coding RNA regulation (Gomes et al., 2013). Therefore, epigenetic regulation constitutes a key regulatory mechanism in the etiology of human complex diseases (Soejima, 2009). Further, high-order epistatic interactions of genes within-/across-pathways, environmental risk factors, and gene-environment interactions contribute to attenuations of genome-trait correlations. Systems biology, the integration of multi-omics techniques, aims at the universal detection of causers for diseases and understanding of newly emerging properties revealed by holistic analyses of high-dimensional multi-omics data. It relies on an interplay between hypothesis- and discovery-driven investigations, and offers significant promises in identifying intermediate causers and causal pathways for complex traits. Existing single-platform association studies, even if helpful and successful for some scenarios, are clearly incompetent to decipher the systems pathology of complex diseases.

In this article, we formulate the central dogma of molecular biology using a multi-omics model. Under this model, we inspect the power of combination of the extreme phenotype sampling scheme and the widely-used *t*-test, providing power and sample size analyses. These results would be helpful to design cost-effective studies as well as to develop novel multi-stage causal association inference methods. As proven, detecting associations between the more proximal variables (protein and gene expression) and the trait is more powerful than detecting the genotype-trait association. Thus, it would be effective to unravel the unidirectional causal pathways from DNA to the endpoint trait using a multi-stage strategy. The first stage identifies candidate proteins by testing trait-protein associations. The second stage identifies candidate RNAs for each candidate protein identified at the first stage by testing protein-RNA associations. The third stage identifies candidate SNPs for each RNA identified at the second stage by testing RNA-SNP associations. Each stage would remove massive non-significant variables and thus essentially reduce multiple testing burden. This strategy would effectively identify candidate genetic instruments and vertical pleiotropy pathways for further causal inferences, i.e., Mendelian randomization inferences (Smith and Ebrahim, 2003; Davey Smith and Hemani, 2014). We acknowledge that reverse causations would exist although we do not model them herein. For example, over-abundance of protein may trigger reduction in mRNA through signaling mechanism. It is instructive to examine reverse causal effects and inspect performance of existing modern multi-omics methods under extreme sampling. Systems biology offers promises in identifying intermediate causers as well as unidirectional and multidirectional causal pathways. Innovative graphical inference methods and efficient computational toolkits are in crucial demands to holistically exploit high-dimensional multi-omics data.

## Author Contributions

HQ conceived the project, prepared ground theoretical results and conducted numerical explorations. HQ and TN wrote the manuscript. JZ contributed constructive comments. All authors read and approved the final manuscript.

### Conflict of Interest Statement

The authors declare that the research was conducted in the absence of any commercial or financial relationships that could be construed as a potential conflict of interest.
