# Supplementary material for: Identifying Multi-Omics Causers and Causal Pathways for Complex Traits
Source: Front Genet. 2019 Feb 21;10:110. doi: 10.3389/fgene.2019.00110 (PMC6393387; doi:10.3389/fgene.2019.00110)
Supplement: Supplementary file 1 [file Data_Sheet_1.docx]

Supplementary Material

**Appendix A. Basic models and properties**

1. **A simple additive genetic model**

Consider the additive genetic model (AGM)$\eta=Gb+\varepsilon$*,* where $G\sim\mathcal{B}\left( 2,p \right)$ stands for the genotypic score (copy number of the minor allele) at a causal SNP, and $p\in(0, 0.5)$ stands for the minor allele frequency (MAF) at the SNP, and $\varepsilon\sim\mathcal{N}\left( 0,1 \right)$ stands for random error that is independent of $G$. Let $\phi\left( \cdot\right)$ and $\Phi\left( \cdot\right)$ stand for the probability density function (PDF) and the cumulative distribution function (CDF) of $\varepsilon$, respectively. For each $\alpha\in(0,0.5)$, let $\varepsilon_{\alpha}$ and $\varepsilon_{1-\alpha}$ stand for the lower and upper quantiles of $\varepsilon$, respectively. Let $L_{\eta}\left( y \right)≝\Pr\left( \eta\leq y \right)$ stand for the CDF of $\eta$, and let $U_{\eta}\left( y \right)≝\Pr\left( \eta\geq y \right)=1-L_{\eta}\left( y \right)$ stand for the upper tail probability (UTP) of $\eta$. Let $E_{G}\left( \cdot\right)$ and $V_{G}\left( \cdot\right)$ denote the expectation and variance of a function of $G$, respectively.

**Lemma 1 (Basic properties of the AGM):** (**i**) *For each pair of* $p\in(0,0.5)$ *and finite* $b\in\left( -\infty,\infty\right)$*,* *the PDF of* $\eta$ *is given by* $l_{\eta}\left( y \right)=E_{G}\left[ \phi\left( y-Gb \right) \right]$ *for* $\forall y\in\left( -\infty,\infty\right).$ (**ii**) *There exist unique real numbers* $\left\{ \eta_{\alpha}, \eta_{1-\alpha} \right\}$ *s.t.* $L_{\eta} \left( \eta_{\alpha} \right)=U_{\eta} \left( \eta_{1-\alpha} \right)=\alpha$ *for each set of* $\alpha\in\left( 0,0.5 \right)$*,* $p\in(0,0.5)$ *and finite* $b\in\left( -\infty,\infty\right)$*.* (**iii**) *For each pair of*$\alpha\in\left( 0,0.5 \right)$ *and* $p\in(0,0.5)$*, the equations* $L_{\eta} \left( \eta_{\alpha} \right)=\alpha$ *and* $U_{\eta} \left( \eta_{1-\alpha} \right)=\alpha$ *define* $\eta_{\alpha}$ *and* $\eta_{1-\alpha}$ *as two functions that are rigorously ascending w.r.t.* $b\in\left( -\infty,\infty\right)$*.* (**iv**) *For each pair of* $\alpha\in(0,0.5)$ *and* $p\in(0,0.5)$*,* *the difference* $\Delta_{\eta}≝\eta_{1-\alpha}-\eta_{\alpha}$ *is* *rigorously descending w.r.t.* $b\in\left( -\infty,0 \right]$*, is rigorously ascending w.r.t.* $b\in\left[ 0,\infty\right)$*, and thus achieves minimum value* $\Delta_{\varepsilon}≝\varepsilon_{1-\alpha}-\varepsilon_{\alpha}$ *at the unique minimizer* $b=0$*.* (**v**) *For each* $\alpha\in(0,0.5)$ *and each k, the conditional moments* $\mu_{k,G|0}≝E\left( G^{k}|\eta<\eta_{\alpha} \right)$ *and* $\mu_{k,G|1}≝E\left( G^{k}|\eta>\eta_{1-\alpha} \right)$ *are respectively formulated by* $\mu_{k,G|0}=E_{G}\left[ G^{k}\Phi\left( \eta_{\alpha}-G\beta\right) \right]$ *and* $\mu_{k,G|1}=E_{G}\left[ G^{k}\bar{\Phi}\left( \eta_{1-\alpha}-G\beta\right) \right]$*.*

**Remark 1:** Under the AGM, if $b=0$, then $\eta=\varepsilon\sim\mathcal{N}\left( 0,1 \right)$ uniformly for $\forall p\in(0,0.5)$. For each pair of $b\neq0$ and $p\in(0,0.5)$*,* the PDF of $\eta$ is mixed from PDFs of three normal densities $\phi\left( y \right)$, $\phi\left( y-b \right)$ and $\phi\left( y-2b \right)$ with respective mixture weights $w_{0}=\left( 1-p \right)^{2}$, $w_{1}=2p(1-p)$ and $w_{2}=p^{2}$. The model coefficient $b$ determines the locations of the three peaks of the mixed density, and the MAF $p$ determines the areas under these peaks.

**Remark 2:** For each pair of finite $b\in\left( -\infty,\infty\right)$ and$p\in\left( 0,0.5 \right)$, the CDF $L_{\eta}\left( y \right)$ is a bounded $0<L_{\eta}\left( y \right)<1$ for $\forall y\in\left( -\infty,\infty\right)$ and rigorously ascending function w.r.t. $y\in\left( -\infty,\infty\right)$. Hence, for a specific$\alpha\in\left( 0,0.5 \right)$*,* there exists a unique threshold $\eta_{\alpha}$ such that $L_{\eta}\left( \eta_{\alpha} \right)=\alpha$. The UTP $U_{\eta}\left( y \right)$ is a bounded $0<U_{\eta}\left( y \right)<1$ for $\forall y\in\left( -\infty,\infty\right)$ and rigorously descending function w.r.t. $y\in\left( -\infty,\infty\right)$. Hence, for a specific$\alpha\in\left( 0,0.5 \right)$, there exists a unique real number $\eta_{1-\alpha}$ such that $U_{\eta}\left( \eta_{1-\alpha} \right)=\alpha$. In particular, if $b=0$, then $\eta=\varepsilon\sim\mathcal{N}\left( 0,1 \right)$, $\eta_{1-\alpha}=\varepsilon_{1-\alpha}$ and $\eta_{\alpha}=\varepsilon_{\alpha}$ uniformly for $\forall p\in(0,0.5)$. For given truncation level $\alpha$, MAF $p$ and effect size $b$, the truncation thresholds $\eta_{1-\alpha}$ and $\eta_{\alpha}$ can be numerically evaluated according to the monotonicity of $L_{\eta}(y)$ and $U_{\eta}(y)$ w.r.t. $y$.

**Remark 3:** For each pair of $\alpha\in\left( 0,0.5 \right)$ and $p\in\left( 0,0.5 \right)$*,* the equation $L_{\eta} \left( \eta_{\alpha} \right)=\alpha$ defines $\eta_{\alpha}$ an implicit function of $b\in\left( -\infty,\infty\right)$, and the equation $U_{\eta} \left( \eta_{1-\alpha} \right)=\alpha$ defines $\eta_{1-\alpha}$ as another implicit function of $b\in\left( -\infty,\infty\right)$. The monotonicity of $\eta_{\alpha}$ and $\eta_{1-\alpha}$ is warranted by $\partial\eta_{1-\alpha}/\partial b>0$ and $\partial\eta_{\alpha}/\partial b>0$ for $\forall b\in(-\infty,\infty)$. The definite positivity of $\partial\eta_{1-\alpha}/\partial b$ and $\partial\eta_{\alpha}/\partial b$, as can be mathematically proven, holds uniformly for all sets of $\alpha\in\left( 0,0.5 \right)$, $p\in\left( 0,0.5 \right)$ and $b\in\left( -\infty,\infty\right)$.

**Remark 4:** For each pair of $\alpha\in\left( 0,0.5 \right)$ and$p\in\left( 0,0.5 \right)$, the difference $\Delta_{\eta}=\eta_{1-\alpha}-\eta_{\alpha}$ appears rigorously descending w.r.t. $b\in(-\infty,0]$ and appears rigorously ascending w.r.t. $b\in[0, \infty)$*.* This property is warranted by $\partial\Delta_{\eta}=\partial\eta_{1-\alpha}/\partial b-\partial\eta_{\alpha}/\partial b<0$ for $b\in(-\infty,0)$*,* $\partial\Delta_{\eta}/\partial b>0$ for $b\in(0,\infty)$*,* and $\left. \partial\Delta_{\eta}/\partial b \right|_{b=0}=0$. Therefore, the difference $\Delta_{\eta}$ approaches minimum value $\Delta_{\varepsilon}=\varepsilon_{1-\alpha}-\varepsilon_{\alpha}$ at the unique minimizer $b=0$.

1. **A** **mediate causal variable model**

Consider a single mediate causal variable model (SMCVM) as depicted in **Supplementary Figure 1:** $\{Y=X\beta+e$, $X=G\gamma+u$}, where $X$ is the mediate causal variable, $G\sim\mathcal{B}(2,p$) is the genotypic score at a causal SNP with MAF $p\in(0,0.5)$, and the exogenous Gaussian errors $e\sim\mathcal{N}\left( 0, \sigma_{e}^{2} \right)$ and $u\sim\mathcal{N}\left( 0, \sigma_{u}^{2} \right)$ are independent of each other. Under this model, $G$ is called the indirect causal variable and $X$ is called the direct causal variable for outcome variable $Y$.

**Supplementary Figure 1.** **Causal model with a single mediate causal variable.** The indirect causal variable $G$ impacts the distribution of $Y$ via mediate causal variable $X$ only. The exogenous random errors $u$ and $e$ are independent of each other.

The entire parameter space of the SMCVM is $\boldsymbol{\Omega}=\{ \boldsymbol{\omega}=\left( \beta,\gamma,p,\sigma_{e}^{2},\sigma_{u}^{2} \right):-\infty<\beta,\gamma<\infty,0<p<0.5,$and$0<\sigma_{e}^{2},\sigma_{u}^{2}<\infty\}$. For each $\boldsymbol{\omega\in}\boldsymbol{\Omega}$, let $L_{Y}\left( y \right)≝\Pr\left( Y\leq y \right)$ stand for the CDF of $Y$, and let $U_{Y}\left( y \right)≝\Pr\left( Y\geq y \right)=1-L_{Y}\left( y \right)$ stand for the UTP of $Y$. Let $E_{X}\left( \cdot\right)$ and $V_{X}\left( \cdot\right)$ denote the expectation and variance of a function of $X$, respectively.

**Lemma 2 (Basic properties of the SMCVM):**

(**i**) *The first 4 moments of the mediate causal variable* $X$ *are given by:*

$$\mu_{1,X}=\gamma\mu_{1,G},$$

$$\mu_{2,X}=\gamma^{2}\mu_{2,G}+\mu_{2,u},$$

$$\mu_{3,X}=\gamma^{3}\mu_{3,G}+3\gamma\mu_{1,G}\mu_{2,u},$$

$$\mu_{4,X}=\gamma^{4}\mu_{4,G}+6\gamma^{2}\mu_{2,G}\mu_{2,u}+\mu_{4,u},$$

*where* $\mu_{2,u}=\sigma_{u}^{2}$*,* $\mu_{4,u}=3\sigma_{u}^{4}$*,* $\mu_{1,G}=2p$*,*$\mu_{2,G}=2p(1+p)$*,*$\mu_{3,G}=2p\left( 1+3p \right)$*, and* $\mu_{4,G}=2p\left( 1+7p \right)$*. The variance of X is given by* $\sigma_{X}^{2}=\mu_{2}\left( X \right)-\mu_{1}^{2}\left( X \right)$*.*

(**ii**) *The PDFs of* $X$ *and Y are given by* $l_{X}\left( x \right)=\sigma_{u}^{-1}E_{G}\left[ \phi\left( \left( x-G\gamma\right)/\sigma_{u} \right) \right]$ *and* $l_{Y}\left( y \right)=\sigma_{v}^{-1}E_{G}\left[ \phi\left( \left( y-G\beta\gamma\right)/\sigma_{v} \right) \right]$*, respectively, where* $\sigma_{v}=\sqrt{{\sigma_{e}^{2}+\beta^{2}\sigma}_{u}^{2}}$ *is the standard deviation of* $v=e+\beta u$*.*

(**iii**) *For each* $\alpha\in\left( 0,0.5 \right)$ *and each* $\boldsymbol{\omega\in}\boldsymbol{\Omega}$*, there exist unique lower and upper* $\alpha$ *truncation thresholds* $\left\{ Y_{\alpha}, Y_{1-\alpha} \right\}$ *such that* $L_{Y} \left( Y_{\alpha} \right)=U_{Y} \left( Y_{1-\alpha} \right)=\alpha$*.*

(**iv**) *For each set of* $\alpha\in(0,0.5)$*,* $p\in(0,0.5)$*,* $\sigma_{e}^{2}\in(0, \infty)$*,* $\sigma_{u}^{2}\in(0,\infty)$*, and finite* $\gamma\neq0$ *(no matter* $>0$ *or* $<0$*), the lower truncation threshold* $Y_{\alpha}$ *is rigorously ascending w.r.t.* $\beta\in(-\infty,0]$ *and is rigorously descending w.r.t.* $\beta\in[0,\infty)$*. In contrast, the upper truncation threshold* $Y_{1-\alpha}$ *is rigorously descending w.r.t.* $\beta\in(-\infty,0]$ *and is rigorously ascending w.r.t.* $\beta\in[0,\infty)$*.*

(**v**) *For each set of* $\alpha\in(0,0.5)$*,* $p\in(0,0.5)$*,* $\sigma_{e}^{2}\in(0, \infty)$*,* $\sigma_{u}^{2}\in(0,\infty)$*, and finite* $\gamma\neq0$ *(no matter* $>0$ *or* $<0$*), the difference* $\Delta_{Y}≝Y_{1-\alpha}-Y_{\alpha}$ *is rigorously descending w.r.t.* $\beta\in\left( -\infty,0 \right]$*, rigorously ascending w.r.t.* $\beta\in\left[ 0,\infty\right)$*, and thus achieves minimum value* $\Delta_{e}=\left( \varepsilon_{1-\alpha}-\varepsilon_{\alpha} \right)\sigma_{e}$ *at the unique minimizer* $\beta=0$*.*

(**vi**) *For each* $\alpha\in(0,0.5)$ *and each* $\boldsymbol{\omega\in}\boldsymbol{\Omega}$*, the conditional PDF*  of $X$ given $Y\leq Y_{\alpha}$ is

$$f_{X|Y\leq Y_{\alpha}}\left( x \right)=\frac{l_{X}\left( x \right)}{\alpha}\Phi\left( \frac{Y_{\alpha}-x\beta}{\sigma_{u}} \right),\forall x\in(-\infty,\infty);$$

*and the conditional PDF of* $X$ *given* $Y\geq\tau_{1-\alpha}$ *is*

$$f_{X|Y\geq Y_{1-\alpha}}\left( x \right)=\frac{l_{X}\left( x \right)}{\alpha}\bar{\Phi}\left( \frac{Y_{1-\alpha}-x\beta}{\sigma_{u}} \right),\forall x\in(-\infty,\infty).$$

**(vii)** *For* $\forall\alpha\in(0,0.5)$*, the* $k^{th}$*conditional moments* $\mu_{k,X|0}≝E\left( X^{k}|Y\leq Y_{\alpha} \right)$ *and* $\mu_{k,X|1}≝E\left( X^{k}|Y\geq Y_{1-\alpha} \right)$ *are formulated by*

$$\mu_{k,X|0}=\int x^{k}\frac{l_{X}\left( x \right)}{\alpha}\Phi\left( \frac{Y_{\alpha}-x\beta}{\sigma_{e}} \right)dx$$

*and*

$$\mu_{k,X|1}=\int x^{k}\frac{l_{X}\left( x \right)}{\alpha}\bar{\Phi}\left( \frac{Y_{1-\alpha}-x\beta}{\sigma_{e}} \right)dx,$$

*respectively. Let* $T=exp \left( cX \right)$ *for an arbitrary constant* $c>0$*. The conditional means* $\mu_{T|0}≝E\left( T|Y\leq Y_{\alpha} \right)$ *and* $\mu_{T|1}≝E\left( T|Y\geq Y_{1-\alpha} \right)$ *are formulated by*

$$\mu_{T|0}=\int\frac{K\left( x \right)}{\alpha}\Phi\left( \frac{Y_{\alpha}-x\beta}{\sigma_{e}} \right)dx$$

*and*

$$\mu_{T|1}=\int\frac{K\left( x \right)}{\alpha}\bar{\Phi}\left( \frac{Y_{1-\alpha}-x\beta}{\sigma_{e}} \right)dx,$$

*respectively, where*

$$K\left( x \right)=\frac{1}{\sigma_{u}}\exp\left( \frac{c^{2}\sigma_{u}^{2}}{2} \right)E_{G}\left[ \phi\left( \frac{x-G\gamma}{\sigma_{u}}-c\sigma_{u} \right)\exp\left( cG\gamma\right) \right].$$

**(viii)** *For each set of* $\alpha\in(0,0.5)$*,* $p\in(0,0.5)$*,* $\sigma_{e}^{2}\in(0, \infty)$*,* $\sigma_{u}^{2}\in(0,\infty)$*, and finite* $\gamma\in(-\infty,\infty)$*, the conditional means* $\mu_{T|1}$ *and* $\mu_{T|0}$ *are infinitely differentiable w.r.t.* $\beta\in(-\infty,\infty)$*, and so is the fold change* $R≝\mu_{T|1}/\mu_{T|0}$*;* $\mu_{T|1}$ *is rigorously ascending w.r.t.* $\beta\in(-\infty,\infty)$*,* $\mu_{T|0}$ *is rigorously descending w.r.t.* $\beta\in(-\infty,\infty)$*, and thus* $R$ *is rigorously ascending w.r.t.* $\beta\in(-\infty,\infty)$*.*

**Remark 5:** Under the SMCVM, we can rewrite $Y=G\theta+v$, where $\theta=\beta\gamma$, and $v=e+\beta u\sim\mathcal{N}\left( 0,\sigma_{v}^{2} \right)$ is independent of $G\sim\mathcal{B}\left( 2,p \right)$. Define $\eta=Y/\sigma_{v}$, $b=\theta/\sigma_{v}$, and $\varepsilon=v/\sigma_{v}$. Then, $\eta=Gb+\varepsilon$, where $\varepsilon\sim\mathcal{N}\left( 0,1 \right)$ is independent of $G\sim\mathcal{B}\left( 2,p \right)$. For each set of fixed $\alpha,\gamma,p,\sigma_{e}^{2}$ and $\sigma_{u}^{2}$, the lower and upper truncation thresholds $\{\eta_{\alpha},\eta_{1-\alpha}\}$ are implicit functions w.r.t. $\beta\in\left( -\infty,\infty\right)$. If $\gamma>0$, then $\{\eta_{\alpha},\eta_{1-\alpha}\}$ are rigorously ascending w.r.t. $\beta\in\left( -\infty,\infty\right)$. If $\gamma<0$, then $\{\eta_{\alpha},\eta_{1-\alpha}\}$, are rigorously descending w.r.t. $\beta\in\left( -\infty,\infty\right)$. If $\gamma=0$, then $\{\eta_{\alpha},\eta_{1-\alpha}\}$, does not change w.r.t. $\beta\in\left( -\infty,\infty\right)$. The difference $\Delta_{\eta}=\eta_{1-\alpha}-\eta_{\alpha}$ approaches minimum value $\Delta_{\varepsilon}=\varepsilon_{1-\alpha}-\varepsilon_{\alpha}$ at the unique minimizer $b=0$.

**Remark 6:** The CDF $L_{Y}\left( y \right)≝\Pr\left( Y\leq y \right)$ rigorously increases w.r.t. $y\in\left( -\infty,\infty\right)$, and the UTP $U_{Y}\left( y \right)≝\Pr\left( Y\geq y \right)=1-\Pr\left( Y\leq y \right)=1-L_{Y}\left( y \right)$ rigorously decreases w.r.t. $y\in\left( -\infty,\infty\right)$. The monotonicity of $L_{Y}\left( y \right)$ and $U_{Y}\left( y \right)$ warrants the existence and uniqueness of the truncation thresholds $\left\{ Y_{\alpha}, Y_{1-\alpha} \right\}$ for each truncation level $\alpha\in(0,0.5)$. The lower $\alpha$ truncation threshold of $Y$ is given by $Y_{\alpha}=\sigma_{v}\eta_{\alpha}$, and the upper $\alpha$ truncation threshold of $Y$ is given by $Y_{1-\alpha}=\sigma_{v}\eta_{1-\alpha}$. For each set of fixed $\alpha,\gamma,p,\sigma_{e}^{2}$ and $\sigma_{u}^{2}$, the lower and upper truncation thresholds $\{Y_{\alpha},Y_{1-\alpha}\}$ are implicit functions w.r.t. $\beta\in\left( -\infty,\infty\right)$. No matter is $\gamma$ positive, zero or negative, the lower $\alpha$ truncation threshold $Y_{\alpha}$ appears ascending w.r.t. $\beta\in\left( -\infty,0 \right]$, descending w.r.t. $\beta\in\left[ 0,\infty\right)$, and has unique maximizer $\beta=0$. In contrast, the upper $\alpha$ truncation threshold $Y_{1-\alpha}$ appears descending w.r.t. $\beta\in\left( -\infty,0 \right]$, ascending w.r.t. $\beta\in\left[ 0,\infty\right)$, and has unique minimizer $\beta=0$. Therefore, the difference $\Delta_{Y}≝Y_{1-\alpha}-Y_{\alpha}$ approaches the minimum value $\Delta_{Y}=\left( \varepsilon_{1-\alpha}-\varepsilon_{\alpha} \right)\sigma_{e}$ at the unique minimizer $\beta=0$. Altering the direction of $\gamma$ does not qualitatively change the pattern of the lower $\alpha$ truncation threshold $Y_{\alpha}$ or that of the upper $\alpha$ truncation threshold $Y_{1-\alpha}$.

**Remark 7:** The log2 transformation, explicitly, ${X=log}_{2} (T)$ of intensity $T$, is very popular in expression studies to fit normality. Therefore, $c=log(2)$ is an important example for the constant in **Lemma 2.vii & viii**. Under the SMCVM, for each set of fixed $\alpha,\gamma,p,\sigma_{e}^{2}$ and $\sigma_{u}^{2}$, the truncation means $\mu_{T|1}$ and $\mu_{T|0}$ and the fold change $R=\mu_{T|1}/\mu_{T|0}$ are implicit functions w.r.t. mediate effect size $\beta\in\left( -\infty,\infty\right)$. No matter is $\gamma$ positive, zero or negative, the lower truncation mean and $\mu_{T|0}$ appears descending w.r.t. $\beta$, the upper truncation mean $\mu_{T|1}$ appears ascending w.r.t. $\beta$, and $\mu_{T|1}=\mu_{T|0}$ only at $\beta=0$. Accordingly, the fold change $R$ appears ascending w.r.t. $\beta$. The monotonicity of the fold change can be utilized to numerically compute the mediate effect size $\beta$ for a specific $R$ value. It is clear that that $R=1$ if and only if $\beta=0$.

Let $\left\{ {(Y}_{i},X_{i},G_{i},e_{i},u_{i} \right):i=1,\ldots,n\}$be randomly generated from the SMCVM. Let $\hat{\rho}_{n}$ be the sample coefficient of Pearson correlation between ${(Y}_{1},\ldots,Y_{n})$ and $(X_{1},\ldots,X_{n})$, and let $\hat{r}_{n}$ be the sample coefficient of Pearson correlation between ${(Y}_{1},\ldots,Y_{n})$ and $(G_{1},\ldots,G_{n})$*.* The asymptotic distributions of $\hat{\rho}_{n}$ and $\hat{r}_{n}$ are formulated in **Proposition 1** and **Corollary 1**, respectively. In context, the notation “$≝$” reads as “defined as”, and the notation “$\underset{\to}{d.}$” reads as “converges in distribution to”.

**Proposition 1.** *As sample size* $n\to\infty$*, the statistic* $t_{n}^{2}≝n\hat{\rho}_{n}^{2}/(1-\hat{\rho}_{n}^{2})\underset{\to}{d.}\lambda^{2}\chi_{1,n\tau^{2}}^{2}$*, where*

$$\lambda^{2}=1+\frac{\beta^{2}}{\sigma_{e}^{2}}\left( 6\mu_{1,X}^{2}+\frac{3\mu_{1,X}^{4}-4\mu_{1,X}\mu_{3,X}+\mu_{4,X}}{\sigma_{X}^{2}}-\sigma_{X}^{2} \right) and \tau^{2}=\frac{\beta^{2}\sigma_{X}^{2}}{\lambda^{2}\sigma_{e}^{2}}.$$

*In particular,* $t_{n}^{2}\underset{\to}{d.}\chi_{1}^{2}$ *when* $n\to\infty$*, if the null hypothesis* $H_{0X}:\beta=0$ *is true.*

**Corollary 1.** *The statistic* $s_{n}^{2}\underset{\to}{d.}\lambda_{0}^{2}\chi_{1,n\tau_{0}^{2}}^{2}$ *when* $n\to\infty$*, where* $\lambda_{0}^{2}=1+\left( 1-2pq \right)\theta^{2}/\sigma_{v}^{2}$*,* $\tau_{0}^{2}=2pq\theta^{2}/(\sigma_{v}^{2}\lambda_{0}^{2})$*,* $q=1-p$*,* $\theta=\beta\gamma$*, and* $\sigma_{v}^{2}=\sigma_{e}^{2}+\beta^{2}\sigma_{u}^{2}$*. In particular,* $s_{n}^{2}\underset{\to}{d.}\chi_{1}^{2}$ *when* $n\to\infty$*, if the null hypothesis* $H_{0G}:\theta=0$ *is true.*

**Remark 8:** The standard correlation test statistic is$t_{n}^{*2}=(1-2/n)t_{n}^{2}$. If $(X,Y)$ follows a bivariate normal distribution of zero correlation, then $t_{n}^{*2}\sim F_{1,n-2}$ for finite $n>2$. Beyond the joint normality assumption, e.g., under the SMCVM, the finite sample distribution of $t_{n}^{2}$ is generally intractable. If $X$ and $Y$ are independent, then $t_{n}^{2}\underset{\to}{d.}\chi_{1}^{2}$ when $n\to\infty$.

**Remark 9:** The generic asymptotic distribution in **Proposition 1** can be utilized to numerically compute the type I error rate and statistical power of the test for the correlation between trait value $Y$ and mediate causal variable $X$. For a given nominal significance level $NSL\in(0,0.5)$, the rejection threshold can be derived as the upper $\mathrm{NSL}$ quantile $q_{1-NSL}$ of the chi-square distribution with 1 degree of freedom $\chi_{1}^{2}$: $\Pr\left( \chi_{1}^{2}>q_{1-NSL} \right)=\mathrm{NSL}$. The power function of the correlation test is given by $pwr=Pr\left( \chi_{1,n\tau^{2}}^{2}>q_{1-NSL}/\lambda^{2} \right)$ for $\forall\beta\in\left( -\infty,\infty\right)$. Both the $q_{1-NSL}$ and the $\mathrm{pwr}$ can be numerically computed by the built-in R functions $\mathrm{qchisq}\left( \cdots\right)$ and $\mathrm{pchisq}\left( \cdots\right)$.

**Remark 10:** Similarly, the generic asymptotic distribution in **Corollary 1** can be utilized to numerically compute the type I error rate and statistical power of the test for the association between trait value $Y$ and genotype$G$. For the given $NSL\in(0,0.5)$, the rejection threshold is the upper $\mathrm{NSL}$ quantile $q_{1-NSL}$ such that $\Pr\left( \chi_{1}^{2}>q_{1-NSL} \right)=\mathrm{NSL}$, and the power function is given by $pwr=Pr\left( \chi_{1,n\tau_{0}^{2}}^{2}>q_{1-NSL}/\lambda_{0}^{2} \right)$ for $\forall\theta=\beta\gamma\in\left( -\infty,\infty\right)$.

**Proposition 2.** *For* $\alpha\in(0,0.5)$*, let* $\left\{ \left( Y_{0i},X_{0i},G_{0i},u_{0i},e_{0i} \right) \right\}:i=1,\ldots,m\}$*be randomly sampled from the lower* $\alpha$ *tail of the phenotypic distribution, and let* $\left\{ \left( Y_{1i},X_{1i},G_{1i},u_{1i},e_{1i} \right) \right\}:i=1,\ldots,m\}$*be randomly sampled from the upper* $\alpha$ *tail of the phenotypic distribution. Define* $t_{m}^{2}=m\left( \bar{X}_{1}-\bar{X}_{0} \right)^{2}/(S_{1}^{2}+S_{0}^{2})$*, where* and $\bar{X}_{j}=\frac{1}{m}\sum_{i=1}^{m} X_{ji}$ *and* $S_{j}^{2}=\frac{1}{m}{\sum_{i=1}^{m} \left( X_{ji}-\bar{X}_{j} \right)}^{2}$ for $j=0,1.$ *The statistic* $t_{m}^{2}\underset{\to}{a.d.}\chi_{1,m\delta^{2}}^{2}$ *as m*$\to\infty$*, where* $\delta^{2}=\left( \mu_{1,X|1}-\mu_{1,X|0} \right)^{2}/\left( \sigma_{X|1}^{2}+\sigma_{X|0}^{2} \right) ,$ $\sigma_{X|1}^{2}=\mu_{2,X|1}-\mu_{1,X|1}^{2}$, and $\sigma_{X|0}^{2}=\mu_{2,X|0}-\mu_{1,X|0}^{2}$. *In particular, if* $H_{0}:\beta=0$*, then* $t_{m}^{2}\underset{\to}{a.d.}\chi_{1}^{2}$ *as* $n\to\infty$*.*

**Remark 11:** The truncation moments of $X$ are as formulated in **Lemma 2.vi**. The null and general asymptotic distributions of the $t$ statistic presented in **Proposition 2** can be utilized to numerically compute the statistical power of the standard two-sample *t* test. The steps are similar to those in **Remark 9**. First, by the null distribution, we find the upper $\alpha$ quantile $q_{\alpha}$ of $\chi_{1}^{2}$ for a given significant level $\alpha$, using the built-in R function $\mathrm{qchisq}\left( \cdots\right)$. Next, we numerically compute the truncation moments $\mu_{k,X|1}$ and $\mu_{k,X|0}$ ($k=1,2$), using built-in R function $\mathrm{integrate}\left( \cdots\right)$. After obtaining $\delta^{2}$, we compute the power of the two-sample $t$ test as $pwr=Pr\left( \chi_{1,m\delta^{2}}^{2}>q_{\alpha} \right)$, using the built-in R function $\mathrm{pchisq}\left( \cdots\right)$.

**Appendix B. Technical Proofs**

**Proof of Proposition 1:**

The statistic $t_{n}^{2}$ has the following representation:

$$t_{n}^{2}=\frac{\left( \sqrt{n}\hat{\sigma}_{Y,X} \right)^{2}}{\hat{\sigma}_{Y}^{2}\hat{\sigma}_{X}^{2}-\hat{\sigma}_{Y,X}^{2}}, (4)$$

where $\hat{\sigma}_{Y,X}=\frac{1}{n}\sum_{i=1}^{n} \left( Y_{i}-\bar{Y} \right)\left( X_{i}-\bar{X} \right),$ $\hat{\sigma}_{Y}^{2}=\frac{1}{n}\sum_{i=1}^{n} \left( Y_{i}-\bar{Y} \right)^{2},$ $\hat{\sigma}_{X}^{2}=\frac{1}{n}{\sum_{i=1}^{n} \left( X_{i}-\bar{X} \right)}^{2},$ $\bar{Y}=\frac{1}{n}\sum_{i=1}^{n} Y_{i}$, and $\bar{X}=\frac{1}{n}\sum_{i=1}^{n} X_{i}$. Substituting equations $Y_{i}=X_{i}\beta+e_{i}$ ($i=1,\ldots,n$) into the definition of $\hat{\sigma}_{Y,X}$, we derive

$$\hat{\sigma}_{Y,X}=\frac{1}{n}\sum_{i=1}^{n} \left[ \beta\left( X_{i}-\bar{X} \right)+\left( e_{i}-\bar{e} \right) \right]\left( X_{i}-\bar{X} \right)$$

$$=\beta\left[ \frac{1}{n}\sum_{i=1}^{n} {X_{i}^{2}-\left( \bar{X} \right)}^{2} \right]+\frac{1}{n}\sum_{i=1}^{n} X_{i}e_{i}-\bar{X}\bar{e}. (5)$$

Since $\sigma_{X}^{2}=E\left( X^{2} \right)-E^{2}\left( X \right)$, it follows from eq. (5) that

$$\begin{matrix} \hat{\sigma}_{Y,X}=\beta\left[ \frac{1}{n}\sum_{i=1}^{n} \left( X_{i}^{2}-E\left( X^{2} \right) \right)+\sigma_{X}^{2}+\left( E^{2}\left( X \right)-\left( \bar{X} \right)^{2} \right) \right] \\ +\frac{1}{n}\sum_{i=1}^{n} X_{i}e_{i}-E\left( X \right)\bar{e}-\left( \bar{X}-E\left( X \right) \right)\bar{e} \end{matrix}$$

$$\begin{matrix} =\beta\sigma_{X}^{2}+\frac{\beta}{n}\sum_{i=1}^{n} \left( X_{i}^{2}-E\left( X^{2} \right) \right)-\left( \beta\left( \bar{X}+E\left( X \right) \right)+\bar{e} \right)\left( \bar{X}-E\left( X \right) \right) \\ +\frac{1}{n}\sum_{i=1}^{n} X_{i}e_{i}-E\left( X \right)\bar{e}. (6) \end{matrix}$$

From eq. (6), we write $\hat{\sigma}_{Y,X}-\beta\sigma_{X}^{2}$ in matrix form and obtain

$$\sqrt{n}\left( \hat{\sigma}_{Y,X}-\beta\sigma_{X}^{2} \right)=\left( \begin{aligned} \begin{aligned} -\beta\left( \bar{X}+\mu_{1} \right)-\bar{e} \\ \beta\\ 1 \end{aligned} \\ -\mu_{1} \end{aligned} \right)^{'}\sqrt{n}\left( \begin{aligned} \begin{matrix} \bar{X}-\mu_{1} \\ \frac{1}{n}\sum_{i=1}^{n} X_{i}^{2}-\mu_{2} \end{matrix} \\ \begin{matrix} \frac{1}{n}\sum_{i=1}^{n} X_{i}e_{i} \\ \bar{e} \end{matrix} \end{aligned} \right)=\boldsymbol{\theta}_{n}^{'}\boldsymbol{z}_{n}. \left( 7 \right)$$

Writing $\boldsymbol{\theta}=\left( -2\beta\mu_{1}, \beta,1,-\mu_{1} \right)^{'}$, we observe that

$$\boldsymbol{\theta}_{n}=\left( \begin{aligned} \begin{aligned} -2\beta\mu_{1} \\ \beta\\ 1 \end{aligned} \\ -\mu_{1} \end{aligned} \right)+\left( \begin{aligned} \begin{aligned} -\beta\left( \bar{X}-\mu_{1} \right)-\bar{e} \\ 0 \\ 0 \end{aligned} \\ 0 \end{aligned} \right)=\boldsymbol{\theta}+\frac{1}{\sqrt{n}}\left( \begin{aligned} \begin{aligned} O_{p}(1) \\ 0 \\ 0 \end{aligned} \\ 0 \end{aligned} \right). \left( 8 \right)$$

Namely, $\boldsymbol{\theta}_{n}$ converges in probability to $\boldsymbol{\theta}$ with rate of convergence $1/\sqrt{n}$. In addition, according to standard asymptotic normality theorem, I obtain

$$\boldsymbol{z}_{n}\underset{\to}{a.d.}N_{4}(\boldsymbol{0}, \boldsymbol{\Sigma}), (9)$$

where $\boldsymbol{\Sigma}=cov\left( X,X^{2},Xe,e \right)$ is the variance-covariance matrix of random vector $\left( X,X^{2},Xe,e \right)'$. Under the given conditions, it is straightforward to formulate the $\boldsymbol{\Sigma}$ matrix as below:

$$\boldsymbol{\Sigma}=\left[ \begin{aligned} \begin{matrix} \sigma_{X}^{2} \\ \mu_{3}-\mu_{1}\mu_{2} \\ 0 \end{matrix} \\ 0 \end{aligned}\begin{aligned} \begin{matrix} \mu_{3}-\mu_{1}\mu_{2} \\ \mu_{4}-\mu_{2}^{2} \\ 0 \end{matrix} \\ 0 \end{aligned}\begin{aligned} \begin{matrix} 0 \\ 0 \\ \mu_{2}\sigma_{e}^{2} \end{matrix} \\ \sigma_{e}^{2}\mu_{1} \end{aligned}\begin{aligned} \begin{matrix} 0 \\ 0 \\ \sigma_{e}^{2}\mu_{1} \end{matrix} \\ \sigma_{e}^{2} \end{aligned} \right]. (10)$$

Substituting (8) and (9) into (7), we derive by Slucky’s theorem that

$$\sqrt{n}\left( \hat{\sigma}_{Y,X}-\beta\sigma_{X}^{2} \right)\underset{\to}{a.d.}N\left( 0, \sigma_{*}^{2}\boldsymbol{=}\boldsymbol{\theta}^{\boldsymbol{'}}\boldsymbol{\Sigma}\boldsymbol{\theta} \right). (11)$$

Substituting $\boldsymbol{\theta=}\left( -2\beta\mu_{1}, \beta,1,-\mu_{1} \right)^{'}$and eq. (10) into $\sigma_{*}^{2}=\boldsymbol{\theta}^{\boldsymbol{'}}\boldsymbol{\Sigma}\boldsymbol{\theta}$, we derive

$$\sigma_{*}^{2}=\left( \begin{aligned} \begin{aligned} -2\beta\mu_{1} \\ \beta\\ 1 \end{aligned} \\ -\mu_{1} \end{aligned} \right)^{'}\left[ \begin{aligned} \begin{matrix} \sigma_{X}^{2} \\ \mu_{3}-\mu_{1}\mu_{2} \\ 0 \end{matrix} \\ 0 \end{aligned}\begin{aligned} \begin{matrix} \mu_{3}-\mu_{1}\mu_{2} \\ \mu_{4}-\mu_{2}^{2} \\ 0 \end{matrix} \\ 0 \end{aligned}\begin{aligned} \begin{matrix} 0 \\ 0 \\ \mu_{2}\sigma_{e}^{2} \end{matrix} \\ \sigma_{e}^{2}\mu_{1} \end{aligned}\begin{aligned} \begin{matrix} 0 \\ 0 \\ \sigma_{e}^{2}\mu_{1} \end{matrix} \\ \sigma_{e}^{2} \end{aligned} \right]\left( \begin{aligned} \begin{aligned} -2\beta\mu_{1} \\ \beta\\ 1 \end{aligned} \\ -\mu_{1} \end{aligned} \right)$$

$$=\beta^{2}\left( \begin{matrix} -2\mu_{1} \\ 1 \end{matrix} \right)^{'}\left[ \begin{matrix} \sigma_{X}^{2} & \mu_{3}-\mu_{1}\mu_{2} \\ \mu_{3}-\mu_{1}\mu_{2} & \mu_{4}-\mu_{2}^{2} \end{matrix} \right]\left( \begin{matrix} -2\mu_{1} \\ 1 \end{matrix} \right)+\sigma_{e}^{2}\left( \begin{matrix} 1 \\ -\mu_{1} \end{matrix} \right)^{'}\left[ \begin{matrix} \mu_{2} & \mu_{1} \\ \mu_{1} & 1 \end{matrix} \right]\left( \begin{matrix} 1 \\ -\mu_{1} \end{matrix} \right)$$

$$=\beta^{2}\left( 4\sigma_{X}^{2}\mu_{1}^{2}+4\mu_{1}^{2}\mu_{2}-4\mu_{1}\mu_{3}+\mu_{4}-\mu_{2}^{2} \right)+\sigma_{e}^{2}\sigma_{X}^{2}$$

$$=\left[ 1+\frac{\beta^{2}}{\sigma_{e}^{2}}\left( 4\mu_{1}^{2}+\frac{4\mu_{1}^{2}\mu_{2}-4\mu_{1}\mu_{3}+\mu_{4}-\mu_{2}^{2}}{\sigma_{X}^{2}} \right) \right]\sigma_{e}^{2}\sigma_{X}^{2}=\left[ 1+\frac{\beta^{2}}{\sigma_{e}^{2}}\left( 3\mu_{1}^{2}+\left( \mu_{1}^{2}-\mu_{2} \right)+\frac{\mu_{2}\sigma_{X}^{2}+4\mu_{1}^{2}\mu_{2}-4\mu_{1}\mu_{3}+\mu_{4}-\mu_{2}^{2}}{\sigma_{X}^{2}} \right) \right]\sigma_{e}^{2}\sigma_{X}^{2}=\left[ 1+\frac{\beta^{2}}{\sigma_{e}^{2}}\left( 3\mu_{1}^{2}-\sigma_{X}^{2}+\frac{\mu_{2}\left( \mu_{2}-\mu_{1}^{2} \right)+4\mu_{1}^{2}\mu_{2}-4\mu_{1}\mu_{3}+\mu_{4}-\mu_{2}^{2}}{\sigma_{X}^{2}} \right) \right]\sigma_{e}^{2}\sigma_{X}^{2}$$

$$=\left[ 1+\frac{\beta^{2}}{\sigma_{e}^{2}}\left( 3\mu_{1}^{2}-\sigma_{X}^{2}+\frac{3\mu_{1}^{2}\mu_{2}-4\mu_{1}\mu_{3}+\mu_{4}}{\sigma_{X}^{2}} \right) \right]\sigma_{e}^{2}\sigma_{X}^{2}$$

$$=\left[ 1+\frac{\beta^{2}}{\sigma_{e}^{2}}\left( 6\mu_{1}^{2}+\frac{3\mu_{1}^{4}-4\mu_{1}\mu_{3}+\mu_{4}}{\sigma_{X}^{2}}-\sigma_{X}^{2} \right) \right]\sigma_{e}^{2}\sigma_{X}^{2}$$

$$=\lambda^{2}\sigma_{e}^{2}\sigma_{X}^{2}. (12)$$

Now, we rewrite $t_{n}^{2}$ as

$$t_{n}^{2}=\frac{\sigma_{*}^{2}}{\hat{\sigma}_{Y}^{2}\hat{\sigma}_{X}^{2}-\hat{\sigma}_{Y,X}^{2}}\left( \frac{\sqrt{n}\left( \hat{\sigma}_{Y,X}-\beta\sigma_{X}^{2} \right)}{\sqrt{\sigma_{*}^{2}}}+\frac{\sqrt{n}\beta\sigma_{X}^{2}}{\sqrt{\sigma_{*}^{2}}} \right)^{2}≝\lambda_{n}^{2}\left( z_{n}^{*}+\mu_{n}^{*} \right)^{2}. \mathbf{(}13\mathbf{)}$$

According to the law of large number and Slusky’s theorem, we have

$$\underset{n\to\infty}{\mathrm{plim}} \lambda_{n}^{2}=\frac{\sigma_{*}^{2}}{\sigma_{Y}^{2}\sigma_{X}^{2}-\sigma_{Y,X}^{2}}, \mathbf{(}14\mathbf{)}$$

where $\sigma_{Y}^{2}=\mathrm{var}\left( Y \right)=\sigma_{e}^{2}+\beta^{2}\sigma_{X}^{2},$ $\sigma_{X}^{2}=\mathrm{var}\left( X \right)=\gamma^{2}\sigma_{G}^{2}+\sigma_{u}^{2},$and $\sigma_{Y,X}^{2}=\mathrm{cov}^{2}\left( Y,X \right)=\beta^{2}\left( \sigma_{X}^{2} \right)^{2}.$ Substituting these equations and eq. (12) into eq. (14), we obtain

$$\underset{n\to\infty}{\mathrm{plim}} \lambda_{n}^{2}=\frac{\left[ \sigma_{e}^{2}+\beta^{2}\left( 6\mu_{1}^{2}+\frac{3\mu_{1}^{4}-4\mu_{1}\mu_{3}+\mu_{4}}{\sigma_{X}^{2}}-\sigma_{X}^{2} \right) \right]\sigma_{X}^{2}}{\left( \sigma_{e}^{2}+\beta^{2}\sigma_{X}^{2} \right)\sigma_{X}^{2}-\beta^{2}\left( \sigma_{X}^{2} \right)^{2}}$$

$$=\frac{\sigma_{e}^{2}+\beta^{2}\left( 6\mu_{1}^{2}+\frac{3\mu_{1}^{4}-4\mu_{1}\mu_{3}+\mu_{4}}{\sigma_{X}^{2}}-\sigma_{X}^{2} \right)}{\sigma_{e}^{2}+\beta^{2}\sigma_{X}^{2}-\beta^{2}\sigma_{X}^{2}}$$

$$=1+\frac{\beta^{2}}{\sigma_{e}^{2}}\left( 6\mu_{1}^{2}+\frac{3\mu_{1}^{4}-4\mu_{1}\mu_{3}+\mu_{4}}{\sigma_{X}^{2}}-\sigma_{X}^{2} \right)$$

$$=\lambda^{2}. \mathbf{(}15\mathbf{)}$$

From eq. (11) we obtain $z_{n}^{*}\underset{\to}{a.d.}N(0,1)$. From (13) we observe that $s_{n}^{2}$ converges in distribution to $\lambda^{2}\chi_{1,n\tau^{2}}^{2}$, where the non-centrality parameter $n\tau^{2}=\left( \mu_{n}^{*} \right)^{2}.$ From eq. (12) and eq. (15), we observe that $\sigma_{*}^{2}=\lambda^{2}\sigma_{X}^{2}$. Therefore,

$$\tau^{2}=\frac{\beta^{2}\left( \sigma_{X}^{2} \right)^{2}}{\sigma_{*}^{2}}$$

$$=\frac{\beta^{2}\left( \sigma_{X}^{2} \right)^{2}}{\left[ \sigma_{e}^{2}+\beta^{2}\left( 6\mu_{1}^{2}+\frac{3\mu_{1}^{4}-4\mu_{1}\mu_{3}+\mu_{4}}{\sigma_{X}^{2}}-\sigma_{X}^{2} \right) \right]\sigma_{X}^{2}}$$

$$=\frac{\beta^{2}\sigma_{X}^{2}}{\sigma_{e}^{2}+\beta^{2}\left( 6\mu_{1}^{2}+\frac{3\mu_{1}^{4}-4\mu_{1}\mu_{3}+\mu_{4}}{\sigma_{X}^{2}}-\sigma_{X}^{2} \right)}$$

$$=\frac{\frac{\beta^{2}}{\sigma_{e}^{2}}\sigma_{X}^{2}}{1+\frac{\beta^{2}}{\sigma_{e}^{2}} \left( 6\mu_{1}^{2}+\frac{3\mu_{1}^{4}-4\mu_{1}\mu_{3}+\mu_{4}}{\sigma_{X}^{2}}-\sigma_{X}^{2} \right)}$$

$$=\frac{\beta^{2}\sigma_{X}^{2}}{\lambda^{2}\sigma_{e}^{2}}. \mathbf{(}16\mathbf{)}$$

**Proof of Corollary 1:**

This result can be proven along the lines of the proof for **Proposition 1**. Here is an alternative proof. Under the SMCVM, $Y=G\theta+v$, where $\theta=\beta\gamma$, and $v=e+\beta u\sim\mathcal{N}\left( 0,\sigma_{v}^{2} \right)$ is independent of $G\sim\mathcal{B}\left( 2,p \right)$. In words, the SMCVM can be written in a special form where $X=G$ with no error term. In **Proposition 1**, replacing $\beta$ with $\theta$, replacing $\sigma_{e}^{2}$ with $\sigma_{v}^{2}$, and replacing $\mu_{k,X}$’s with $\mu_{k,G}$’s (for $k=1,\ldots,4$), writing $\lambda^{2}$ as $\lambda_{0}^{2}$, and writing $\tau^{2}$ as $\tau_{0}^{2}$, we obtain

$$\lambda_{0}^{2}=1+\frac{\theta^{2}}{\sigma_{v}^{2}}\left( 6\mu_{1,G}^{2}+\frac{3\mu_{1,G}^{4}-4\mu_{1,G}\mu_{3,G}+\mu_{4,G}}{\sigma_{G}^{2}}-\sigma_{G}^{2} \right) and \tau_{0}^{2}=\frac{\theta^{2}\sigma_{G}^{2}}{\lambda_{0}^{2}\sigma_{v}^{2}}.$$

By the moment formulas of genotypic score $G$, the scale parameter $\lambda_{0}^{2}$ can be simplified as below:

$$\lambda_{0}^{2}=1+\frac{\theta^{2}}{\sigma_{v}^{2}}\left( 6\left( 2p \right)^{2}+\frac{3\left( 2p \right)^{4}-4\left( 2p \right)\left( 2p\left( 1+3p \right) \right)+2p\left( 1+7p \right)}{2pq}-2pq \right)$$

$$=1+\frac{\theta^{2}}{\sigma_{v}^{2}}\left( 24p^{2}+\frac{48p^{4}-16p^{2}\left( 1+3p \right)+2p\left( 1+7p \right)}{2pq}-2pq \right)$$

$$=1+\frac{\theta^{2}}{\sigma_{v}^{2}}\left( 24p^{2}+\frac{24p^{3}-8p\left( 1+3p \right)+\left( 1+7p \right)}{q}-2pq \right)$$

$$=1+\frac{\theta^{2}}{\sigma_{v}^{2}}\frac{24p^{2}q+24p^{3}-8p-24p^{2}+\left( 1+7p \right)-2pq^{2}}{q}$$

$$=1+\frac{\theta^{2}}{\sigma_{v}^{2}}\frac{\left( 24p^{2}q+24p^{3}-24p^{2} \right)+\left( 1-p-2pq^{2} \right)}{q}$$

$$=1+\frac{\theta^{2}}{\sigma_{v}^{2}}\frac{24p^{2}\left( q+p-1 \right)+q\left( 1-2pq \right)}{q}$$

$$=1+\frac{\left( 1-2pq \right)\theta^{2}}{\sigma_{v}^{2}}. \left( 17 \right)$$
